# Impact of hypofractionation and tangential beam IMRT on the acute skin reaction in adjuvant breast cancer radiotherapy

**DOI:** 10.1186/s13014-016-0674-y

**Published:** 2016-07-30

**Authors:** Volker Rudat, Alaa Nour, Salam Abou Ghaida, Aziz Alaradi

**Affiliations:** Department of Radiation Oncology, Saad Specialist Hospital, Al Khobar, 31952 Saudi Arabia

**Keywords:** Breast neoplasms, Radiotherapy, IMRT, Dose hypofractionation, Radiation injuries, Risk factors

## Abstract

**Background:**

The purpose of the study was to evaluate the impact of multiple prognostic factors on the acute skin reaction in adjuvant breast cancer radiotherapy, in particular the impact of hypofractionation (HF) compared to conventional fractionation (CF) and tangential beam (TB) IMRT compared to three-dimensional conformal radiotherapy (3DCRT).

**Methods:**

Two-hundred and sixty-six breast cancer patients with postoperative radiotherapy after breast conserving surgery or mastectomy were retrospectively evaluated. Patients were treated with HF (15 fractions of 2.67 Gy; *n* = 121) or CF (28 fractions of 1.8 Gy or 25 fractions of 2.0 Gy; *n* = 145) and TB-IMRT (*n* = 151) or 3DCRT (*n* = 115). The acute skin reactions were prospectively assessed using the CTCAE v4 grading scale. Ordinal regression analysis was used to assess the impact of possible prognostic factors on the maximal acute skin reaction.

**Results:**

Grade 2 skin reactions were observed in 19 % of the patients treated with CF compared to 2 % treated with HF. On univariate analysis, the fractionation regimen, the PTV (breast versus chest wall), the volume of the PTV and the body mass index were significant prognostic factors for the maximum acute skin reaction. On multivariate analysis, the fractionation regimen (*p* < 0.00001) and the volume of the PTV (*p* = 0.0002) remained as independent significant factors.

**Conclusions:**

Our data suggest that HF is associated with a significantly reduced maximal acute skin reaction compared to CF.

## Background

Breast cancer is the most common cancer in women worldwide, and after lung cancer the second most common cancer overall. In 2012, nearly 1.7 million new breast cancer cases were diagnosed representing about 12 % of all new cancer cases and 25 % of all cancers in women [1].

Adjuvant radiotherapy is an important part of breast cancer management. Conventional fractionation regimens (CF) consisting of 25 daily fractions of 2.0 Gy or 28 daily fractions of 1.8 Gy have generally been considered the “standard” adjuvant radiotherapy prescription. Several large, well-conducted randomized trials have established that hypofractionated regimens (HF) such as 15 daily fractions of 2.67 Gy can be equally effective in terms of long-term disease control and late radiation effects compared to the excellent outcomes of more protracted conventional fractionation schedules [2–5]. The HF evaluated in the recent trials were characterized by an increase of the daily fraction dose and a decrease of the total dose at the same time. The reduced number of daily fractions compared to CF results in a benefit to patients and health services in terms of convenience and cost. Most human cancer types respond to the total dose rather than to the size of the daily fractions [6]. In contrast, the late reacting normal tissues respond to the daily fraction size (higher fraction doses increase the risk of late toxicity) and the total dose [7]. The late adverse effects are dose limiting. CF typically use “small” daily fraction doses of 2.0 Gy or 1.8 Gy to deliver the highest possible tolerated total dose, thereby ensuring the highest rate of tumor control. The finding of comparable late toxicity and long-term tumor control with HF (using “higher” fraction doses) compared to CF (using “small” fraction doses) of the recent studies suggests that breast cancer is an exception in showing comparable sensitivity to fraction size as the normal tissues of the breast and ribcage [8].

Like most human cancer types, early reacting normal tissues respond to the total dose rather than to the size of the daily fractions. Due to the lower total dose used with HF compared to CF it can be expected that the acute radiation reactions are lower in patients treated with HF. However, the reports of randomized trials to date have provided little information comparing acute toxic effects with HF as compared with CF [2, 3, 9–11].

Several randomized [12, 13] and retrospective studies [14–16] have suggested that the radiation technique may influence the acute toxic effects in adjuvant breast cancer radiotherapy. Patients treated with intensity modulated radiotherapy (IMRT) showed less acute skin reactions compared to patients treated with standard tangential beam technique using wedge compensation. This observation has been explained with the improvement in the dose distribution homogeneity using breast IMRT compared to standard radiotherapy using wedges [12]. Due to the mathematical form of the linear-quadratic dose effect relationship, hot spots are penalized more severely in a hypofractionated treatment, so-called ‘triple trouble’ [8, 17]. It can therefore be expected that IMRT is in particular beneficial in patients treated with HF. Commonly used beam configurations for breast IMRT are tangential beams (TB-IMRT) or multiple beams from four to seven directions.

Although not a dose limiting factor in breast cancer radiotherapy, acute skin reactions are of clinical importance. Acute skin toxicity affects multiple dimensions of quality of life. They cause physical discomfort, body image disturbance, emotional distress, and impair both day-to-day functioning and satisfaction with radiation treatment [12, 18]. Goal of this study was to evaluate the impact of multiple prognostic factors on the acute skin reaction in breast cancer radiotherapy, in particular the impact of HF compared to CF and TB-IMRT compared to 3DCRT.

## Patients and methods

### Patient population

Two-hundred and sixty-six breast cancer patients with postoperative radiotherapy after breast conserving surgery or mastectomy between March 2014 and April 2016 were evaluated in this study. The acute skin radiation reaction of all patients was prospectively assessed once weekly during and 6 weeks after radiotherapy by two observers using the “dermatitis radiation” grade of the Common Terminology Criteria for Adverse Events (CTCAE v4.03) (Table 1). The dermatitis radiation grade was documented immediately after assessment in the Local Area Network Therapy Information System “Lantis” (Siemens Healthcare, Germany), and a table with all weekly assessments was included in the “End of Treatment Report” of all patients. The two observers were not involved in the statistical analysis of the study. Patient and treatment related data were transferred into a database, anonymized and retrospectively analyzed using specific statistical software. The study was approved by the local institutional ethical committee and conducted in accordance with the Helsinki Declaration in its current version.

**Table 1:** Dermatitis radiation according to the Common Terminology Criteria for Adverse Events v4.03 (CTCAE)

Grade	Description
1	Faint erythema or dry desquamation
2	Moderate to brisk erythema; patchy moist desquamation, mostly confined to skin folds and creases; moderate edema
3	Moist desquamation in areas other than skin folds and creases; bleeding induced by minor trauma or abrasion
4	Life-threatening consequences; skin necrosis or ulceration of full thickness dermis; spontaneous bleeding from involved site; skin graft indicated
5	Death

Patients with the histologically proven diagnosis of breast cancer or breast cancer in situ receiving postoperative radiotherapy of the whole breast after breast conserving surgery or of the chest wall after mastectomy were eligible for the study. Patients with bilateral breast cancer or a history of previous radiotherapy of the chest were excluded from the analysis. Patients were offered a hypofractionated or conventionally fractionated radiotherapy using inverse planned tangential beam intensity modulated radiotherapy (TB-IMRT) or three-dimensional planned conformal radiotherapy using wedge compensation (3DCRT). The decision about the fractionation regimen and radiation technique was based on patient preference. Main factors considered for the choice of the fractionation regimen by the patients were distance to the radiotherapy department, personal commitments limiting the overall treatment time and recommendation of the treating physicians. Patients not or not fully covered by medical insurance tended to opt for 3DCRT for financial reasons. A few patients with unfavourable thoracic geometry and left-sided breast cancer were treated with seven-field IMRT. These patients were not considered in the analysis.

### Fractionation regimen and assessment of the acute skin reaction

The conventional fractionation regimen (CF) for the postoperative radiotherapy of the breast consisted of 28 fractions (fraction dose 1.8 Gy; total dose 50.4 Gy) and for the chest wall of 25 fractions (fraction dose 2.0 Gy; total dose 50.0 Gy). The hypofractionated regimen (HF) for the breast or chest wall consisted of 15 fractions (fraction dose 2.67 Gy; total dose 40.05 Gy). Where indicated, the supraclavicular lymph nodes were treated with the same fractionation regimen used for the breast or the chest wall. Patients were treated once per day and five times per week. If radiation fractions were missed, patients were treated on weekends in order not to exceed the prescribed overall treatment time. Where indicated, an electron boost was applied with five or eight additional fractions with a fraction dose of 2.0 Gy.

For the analysis of the radiation reaction of the skin, the maximal “dermatitis radiation” grade according to CTCAE v4.03 was used observed during the CF or HF of the whole breast or chest wall (before a possible boost to the PTV; for CF at the planned dose of 50.4 Gy or 50.0 Gy, for HF at the planned dose of 40.05 Gy). Likewise, the time to the dermatitis radiation grade 1 or grade 2 was defined as the time in days from the first radiation fraction to the corresponding dermatitis radiation grade observed during the course of the CF of HF of the whole breast or chest wall (before a possible boost to the PTV).

### Treatment planning

A non-contrast CT-simulation was performed in the supine position on a carbon breast board with the ipsilateral arm up and head turned to the contralateral side. Radio-opaque wires were used to mark the clinical boundaries. A CT scan was performed using 5 mm slice thickness. The CT scanning reference point was defined using the CT simulation software Coherence Dosimetrist (Siemens Medical, Germany), and target volumes (PTV and OARs) using the software Coherence Oncologist (Siemens Medical, Germany). The 3DCRT and IMRT plans were generated using the treatment planning system XIO 4.4 (CMS, Inc. of St. Louis, Mo, USA). Two Siemens Oncor Anvantgarde linear accelerators with a 160 MLC Multileaf Collimator were used for the treatment. The leaf width was 0.5 cm at the isocenter. The dose calculation was determined using the “Superposition” algorithm. Dose volume histograms (DVH) of the PTV and OARs of the 3D-CRT and IMRT plans were generated. The target volumes were defined and the dose prescribed according to the International Commission on Radiation Units and Measurement (ICRU) Reports 50 and 62 recommendations. Accordingly, the target volume should be surrounded by the 95 % isodose line of the prescribed dose. The planning target volume (PTV) definition for the whole breast or chest wall was done according to the recommendations of the breast cancer atlas for radiation therapy planning consensus definitions of the Radiation Therapy Oncology Group (RTOG) (http://www.rtog.org/CoreLab/ContouringAtlases/BreastCancerAtlas.aspx). The PTV of the breast included the apparent computed tomography (CT) glandular breast tissue and the PTV of the chest wall the pectoralis muscle, chest wall muscles, and ribs. For the statistical analysis the volume of the PTV (breast or chest wall) was obtained from the dose volume histograms of the 3DCRT or TB-IMRT plans.

Daily online verification and correction of the patient positioning error prior to radiotherapy was performed in all patients using orthogonal megavoltage electronic portal images [19]. No respiratory gating techniques were applied in this study.

### 3DCRT plans

The dose was prescribed to the ICRU reference point which was usually the isocenter located in the PTV volume centroid. Two tangential semi-opposed beams (to avoid divergence), physical wedges (usually 15° or 30°), a 160 MLC Multileaf Collimator and 6 MV photons were used for 3DCRT. A few patients received a mixed beam technique (6 MV and 15 MV photons). The beam angles, wedge angles, and beam weighting (usually minimal) were chosen to optimize coverage of the PTV, while minimizing exposure to the ipsilateral lung, heart and contralateral breast. Gantry angles ranged from 42° to 55° for the medial fields and from 224° to 232° for the lateral fields for patients treated on the right side, and from 305° to 322° for the medial fields and from 133° to 147° for the lateral fields for patients treated on the left side.

### TB-IMRT plans

The same PTV and tangential beam orientation of the 3DCRT plans were used for the TB-IMRT plans. An extension into the air anteriorly of the chest of 1.5 cm was added to the PTV to ensure appropriate opening of the 160 MLC Multileaf Collimator. Inverse treatment planning and 6MV photons were used for all IMRT plans. The dose was prescribed to the PTV, and as initial dose volume constraints the IMRT prescription table provided by the XIO treatment planning system was used (Table 2). The IMRT plans were optimized to cover the PTV and spare the surrounding tissues as much as possible. A step-and-shoot technique was applied. An optimization with 100 iterations was then applied, and followed by a semiautomatic segmentation (minimum 3 cm step size). Segments equal or less than 2 MU were expelled from the plan. Tissue inhomogeneities were considered in the treatment planning optimization process, and the dose calculation algorithm used was “Superposition”. The plans were developed to deliver 95 % of the prescribed dose to the full PTV, and to minimize dose to the OARs lung and heart.

**Table 2:** Patient characteristics stratified by fractionation regimen

Characteristic	Total		Fractionation regimen				*p* value
			CF		HF		
	n	%	n	%	n	%	
Country of origin							
Middle East	154	57.9	89	61.4	65	53.7	0.62
Africa	60	22.6	31	21.4	29	24.0	
Asia	41	15.4	20	13.8	21	17.4	
Europe/U.S.A.	11	4.1	5	3.4	6	5.0	
Age at diagnosis (years)							
Mean	49		49		49		0.85
Standard Deviation	10		9		10		
≤40	53	19.9	29	20.0	24	19.8	0.99
41–50	103	38.7	55	37.9	48	39.7	
51–60	84	31.6	47	32.4	37	30.6	
>60	26	9.8	14	9.7	12	9.9	
Laterality							
Left breast	63	48.5	35	50.7	28	45.9	0.58
Right breast	67	51.5	34	49.3	33	54.1	
Left chest wall	76	55.9	42	55.3	34	56.7	0.87
Right chest wall	60	44.1	34	44.7	26	43.3	
Breast volume (ml)							
Median	1132		1000		1235		0.01
Minimum	285		432		285		
Maximum	3613		2929		3613		
Chest wall volume (ml)							
Median	715		695		776		0.02
Minimum	110		110		230		
Maximum	2521		2521		1715		
BMI							
Median	30.6		29.9		31.4		0.16
Minimum	15.4		15.4		15.7		
Maximum	67.3		66.4		67.3		
<25	44	16.6	19	15.8	25	17.2	0.06
25.0–29.9	73	27.5	25	20.8	48	33.1	
30.0–34.9	80	30.2	46	38.3	34	23.4	
35.0–39.9	46	17.4	19	15.8	27	18.6	
≥40.0	22	8.3	11	9.2	11	7.6	
Menopausal status							
Pre-menopausal	134	50.4	68	46.9	66	54.5	0.21
Post-menopausal	132	49.6	77	53.1	55	45.5	
Histopathology							
Invasive ductal cancer	246	92.5	135	93.1	111	91.7	0.05
Invasive lobular cancer	13	4.9	8	5.5	5	4.1	
DCIS	5	1.9	0	0	5	4.1	
Other	2	0.8	2	1.4	0	0	
T classification							
pTis	6	2.3	0	0.0	6	5.0	0.11
pT0	7	2.6	2	1.4	5	4.1	
pT1	91	34.2	50	34.5	41	33.9	
pT2	104	39.1	59	40.7	45	37.2	
pT3	29	10.9	16	11.0	13	10.7	
pT4	19	7.1	11	7.6	8	6.6	
Not reported	10	3.8	7	4.8	3	2.5	
N classification							
pN0	96	36.1	48	33.1	48	39.7	0.19
pN1	76	28.6	42	29.0	34	28.1	
pN2	56	21.1	28	19.3	28	23.1	
pN3	31	11.7	21	14.5	10	8.3	
Not reported	7	2.6	6	4.1	1	0.8	
M classification							
M0	263	98.9	143	98.6	120	99.2	0.67
M1	3	1.1	2	1.4	1	0.8	
Grading							
G1	22	8.3	5	3.4	17	14.0	0.02
G2	82	30.8	45	31.0	37	30.6	
G3	137	51.5	80	55.2	57	47.1	
Not reported	22	8.3	5	3.4	17	14.0	
ER status							
Negative	65	24.4	36	24.8	29	24.0	0.28
Positive	191	71.8	106	73.1	85	70.2	
Not reported	10	3.8	3	2.1	7	5.8	
PR status							
Negative	15	5.6	7	4.8	8	6.6	0.76
Positive	79	29.7	42	29.0	37	30.6	
Not reported	172	64.7	96	66.2	76	62.8	
Her2/neu status							
Negative	18	6.8	7	4.8	11	9.1	0.39
Positive	178	66.9	99	68.3	79	65.3	
Not reported	70	26.3	39	26.9	31	25.6	

### Statistical analysis

Differences between patient groups treated with CF or HF were assessed using the Chi-square test for categorical variables, the *t*-test for normally distributed and the *U*-test for non-normally distributed continuous variables. All tests were two-sided, and a *p*-value of ≤0.05 was considered significant. To assess the impact of possible prognostic factors on the maximal dermatitis radiation grade an ordinal logistic regression analysis was performed. The model selection of the multivariate analysis was performed by a backward stepwise strategy. The possible prognostic factors included in the analysis are listed in Table 4.

## Results

The patient (Table 2) and treatment characteristics (Table 3) were for the most part well balanced between the patients treated with CF and HF. The volumes of the PTV as well as the body mass index (BMI) were slightly higher in the HF group. Furthermore, 97 % of the patients of the HF group completed their radiotherapy within the prescribed overall treatment time compared to 71 % of the CF group.

**Table 3:** Treatment characteristics stratified by fractionation regimen

	Total		Fractionation regimen				*p* value
			CF		HF		
	n	%	n	%	n	%	
Fractionation regimen							
	266	100.0	145	100.0	121	100.0	
Planning Target Volume (PTV)							
Whole breast	130	48.9	69	47.6	61	50.4	0.65
Chest wall	136	51.1	76	52.4	60	49.6	
Locoregional lymph nodes treated as part of plan							
Yes	133	50.0	77	53.1	56	46.3	0.27
No	133	50.0	68	46.9	65	53.7	
Boost to the PTV							
Yes	129	48.5	83	57.2	46	38.0	<0.01
No	137	51.5	62	42.8	75	62.0	
Radiation technique							
3DCRT	115	43.2	73	50.3	42	34.7	0.01
TB-IMRT	151	56.8	72	49.7	79	65.3	
Prolongation of the prescribed overall treatment time (days)							
0	220	82.7	103	71.0	117	96.7	<0.001
1	20	7.5	18	12.4	2	1.7	
2	13	4.9	12	8.3	1	0.8	
3	6	2.3	5	3.4	1	0.8	
4	5	1.9	5	3.4	0	0	
5	2	08	2	1.4	0	0	
Chemotherapy							
Neo-adjuvant	198	74.4	115	79.3	83	68.6	0.10
Adjuvant	56	21.1	26	17.9	30	24.8	
No chemotherapy	12	4.5	4	2.8	8	6.6	
Hormone therapy							
Yes	198	74.7	111	76.6	87	72.5	0.45
No	67	25.3	34	23.4	33	27.5	
Maximal radiation dermatitis grade (CTC v4.0)^a^							
0	20	7.5	7	4.8	13	10.7	<0.001
1	216	81.2	110	75.9	106	87.6	
2	30	11.3	28	19.3	2	1.7	
Time to dermatitis radiation grade 1 (days)^a^							
Mean	17		21		13		<0.0001
Standard deviation	7		7		3		
Time to dermatitis radiation grade 2 (days)^a^							
Mean	34		35		21		<0.0001
Standard deviation	5		4		1		

*Abbreviation*: ^a^ = Skin reactions observed during the radiotherapy of the whole breast or chest wall (before the possible application of a boost to the PTV; for CF at the planned dose of 50.4 Gy or 50.0 Gy, for HF at the planned dose of 40.05 Gy)

**Fig. 1:**
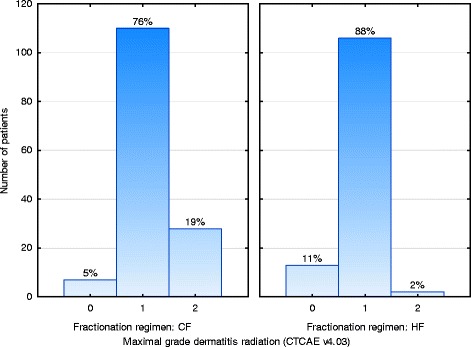
Maximal acute skin reaction stratified by fractionation regimen

**Fig. 2:**
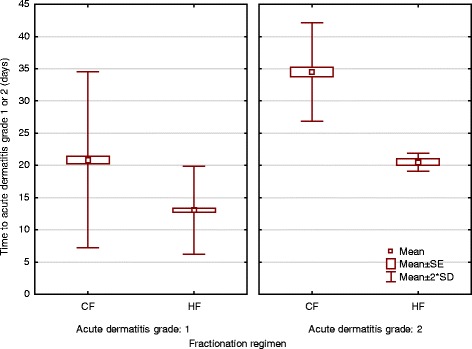
Time to grade 1 and grade 2 acute skin reaction stratified by fractionation regimen

On univariate analysis, the fractionation regimen (Fig. 1), the PTV (breast versus chest wall), the volume of the PTV, and the BMI were significantly associated with the maximal acute skin reaction. On multivariate analysis, only the factors volume of the PTV (*p* = 0.0002) and fractionation regimen (*p* < 0.00001) had a significant independent impact on the maximal acute skin reaction (Table 4). Furthermore, patients treated with HF developed the same acute skin reaction grade significantly earlier compared to patients treated with CF (Table 3, Fig. 2).

**Table 4:** Ordinal logistic regression analysis for maximal grade dermatitis radiation (CTCAE v.4.03)

Factor		Univariate analysis	Multivariate analysis			
		*p* value	*p* value	Estimate	Lower 95 % CI	Upper 95 % CI
Country of origin	Middle East vs. Asia vs. Africa vs. Europe or U.S.A.	0.17				
Age at diagnosis (years)	≤median vs. >median	0.61				
Volume of the PTV (cm^3^)	≤median vs. >median	0.01	0.0002	1.52	0.72	2.32
BMI	<25 vs. 25.0–29.9 vs. 30.0–34.9 vs. 35.0–39.9 vs. ≥40.0	0.03				
Menopausal status	Pre- vs. post-menopausal	0.56				
T classification	Tis, T0,T1,T2 vs. T3,T4	0.51				
N classification	N0 vs. N1, N2, N3	0.28				
Grading	G1, G2 vs. G3	0.61				
ER status	Negative vs. positive	0.98				
PR status	Negative vs. positive	0.79				
Her2/neu status	Negative vs. positive	0.38				
Fractionation regimen	CF vs. HF	<0.0001	<0.00001	2.14	1.32	3.33
Planning Target Volume (PTV)	Breast vs. chest wall	0.02				
Locoregional lymph nodes treated as part of plan	Yes vs. no	0.76				
Radiation technique	3DCRT vs. TB-IMRT	0.42				
Chemotherapy	Neoadjuvant vs. adjuvant vs. no chemotherapy	0.22				
Hormonal therapy	Yes vs. no	0.60				

*Abbreviations*: *95 % CI* = 95 % confidence interval

## Discussion

Our data clearly show that a moderately hypofractionated fractionation regimen (15 daily fractions of 2.67 Gy) results in a significantly less acute skin reaction rate compared to CF in breast cancer radiotherapy.

Two recent randomized [20] and non-randomized studies [21] comparing HF with CF have reported similar results. The randomized study allocated breast cancer patients stage Tis-T2, N0-N1a, M0 (*n* = 287) to adjuvant radiotherapy of the whole breast with either HF (16 daily fractions of 2.67 Gy) or CF (25 daily fractions of 2.0 Gy. Both study arms were followed by a tumor bed boost. Patients were treated with megavoltage tangential portals and forward- or inverse-planned segmental fields in supine or prone position. Acute skin reactions were significantly lower with HF compared to CF (CTCAE v4.0 dermatitis grade 2: 36 % versus 69 %; P_trend_ < 0.001) [20]. The retrospective study compared the acute toxic effects of 2309 patients receiving HF (daily fraction dose >2 Gy (95 % were between 2.6 Gy and 2.7 Gy); mean total dose (SD) 45.3 Gy (2.5 Gy); *n* = 578) versus CF (daily fraction dose ≤2 Gy (62.9 % were 1.8 Gy and 37.1 % were 2.0 Gy); mean total dose (SD) 52.1Gy (2.8 Gy); *n* = 1732) to the whole breast after breast conserving surgery. Sixty percent of the patients treated with HF and 92.9 % of the patients treated with CF received a tumor bed boost. The radiation techniques used were not described in detail in this report. Acute skin reactions were significantly lower with HF compared to CF (CTCAE v4.0 dermatitis grade 2: 28 % versus 61 %; *P* < 0.001) [21]. Our study showed a reduction of the CTCAE 4.0 dermatitis radiation grade 2 from 19 % with CF to 2 % with HF. The lower incidence of grade 2 dermatitis observed in our study compared to the two above studies may be related to differences in the distribution of multiple prognostic factors, for example the total dose. In the above two studies the acute skin reactions were assessed during the radiotherapy of the whole breast and the tumor bed boost whereas in our study only during the radiotherapy to the whole breast or chest wall (before the possible application of a boost). Interestingly, the reported incidence of CTCAE v.2–4 dermatitis radiation grade 2 varies considerably between the studies with CF (9 % [22], 14 % [23], 19 % [this study], 34 % [24], 37 % [12], 61 % [21], 68 % [20] and 68 % [25], 72 % [14]), suggesting that prognostic factors other than the daily fraction dose and total dose significantly influence the acute skin reaction. In our study, the fractionation regimen and the volume of the PTV were identified as the only significant independent prognostic factors for the maximal acute skin reaction grade. The breast volume as prognostic factor for the acute skin reaction has been reported by multiple studies [12, 14, 16, 22, 26–31]. This observation has been explained with the association of large breasts with increased dose inhomogeneity and hot spots [31].

Several studies have reported an impact of the radiation technique on the acute skin reaction [12–16]. IMRT can produce more homogenous dose distributions compared to conventional tangential beam breast cancer radiotherapy using wedge compensation, in particular if compared to two-dimensionally planned breast cancer radiotherapy [12, 32]. However, no significant difference of the superficial dose in breast cancer radiotherapy has been found between tangential beam IMRT (TB-IMRT) and three-dimensional conformal radiotherapy using tangential beams with wedge compensation (3DCRT) in a phantom study [18] and a clinical study using GafChromic film in vivo dosimetry [33]. The superficial dose can be considered as a good surrogate parameter of the skin dose. In agreement with the two in vitro and in vivo dosimetry studies, no significant difference of the acute skin reaction using TB-IMRT compared to 3DCRT was found in our study on univariate and multivariate analysis. An interesting observation in our study is that patients treated with HF developed the same dermatitis radiation grade significantly earlier than the patients treated with CF. The data suggest that the daily fraction size has a significant impact on the time to develop the acute skin reaction, whereas the total dose is more relevant for the severity of the acute skin reaction.

The strength of our study is the uniform prospective assessment of the skin reaction and application of the TB-IMRT and 3DCRT plans by the same team. Limitations of our study are related to the non-randomized study design where a selection bias cannot be excluded with certainty. Furthermore, to obtain the maximal acute skin reaction grade at comparable total doses, the skin reaction during the radiotherapy of the whole breast or chest wall (before the possible application of a boost) was used in the analysis of our study. Some patients may develop their maximal skin reaction after the completion of the radiotherapy of the whole breast or chest wall, and in these patients the maximal skin reaction would have been underestimated. Some possible prognostic factors for the acute skin reaction have not been included in our multivariate analysis, for example genetic markers or smoking during radiotherapy [22, 26, 34].

## Conclusion

Large randomized trials have established that hypofractionated regimens can be equally effective in terms of long-term disease control and late radiation effects compared to conventional fractionation schedules. Our study shows that a moderately hypofractionated regimen is also significantly associated with a reduced maximal acute skin reaction.

## Abbreviations

3DCRT, three-dimensional conformal radiotherapy; BMI, Body mass index; CF, conventional fractionation; CT, computed tomography; CTCAE v4.03, common terminology criteria for adverse events version 4.03; HF, hypofractionationation; PTV, planning target volume; TB-IMRT, inverse planned tangential beam intensity modulated radiotherapy
